# Selected articles from the BioCreative/OHNLP challenge 2018

**DOI:** 10.1186/s12911-019-0994-6

**Published:** 2019-12-27

**Authors:** Sijia Liu, Yanshan Wang, Hongfang Liu

**Affiliations:** Division of Digital Health Sciences, Department of Health Sciences Research, Mayo Clinic, Rochester, MN USA

## Introduction

The wide adoption of electronic health records (EHRs) has led to an improvement in healthcare quality by electronically documenting a patient’s medical conditions, thoughts and actions among the care providers [1]. Those EHR data, with the vast majority being free-texts (e.g., clinical notes, discharge summaries, radiology reports, and pathology reports), have been utilized for primary and secondary purposes, such as documentation need in care process, clinical decision support, outcome improvement, biomedical research and epidemiologic monitoring of the nation’s health. The application of natural language processing (NLP) methods and resources to clinical and biomedical text has received growing attention over the past years, but progress has been limited by difficulties to access shared tools and resources, partially caused by patient privacy and data confidentiality constraints. Efforts to increase sharing and interoperability of the few existing resources are needed to facilitate the progress observed in the general NLP domain. Towards this goal, we organized the BioCreative/OHNLP Challenge 2018 workshop (https://sites.google.com/view/ohnlp2018/home) to promote community efforts on methodological advancements and data curation mechanisms in clinical NLP. The challenge consists of two independent clinical NLP tasks: 1) Family History Extraction; and 2) Clinical Semantic Textual Similarity. The top performing teams were invited to present their solutions during the BioCreative/OHNLP Challenge 2018 workshop in conjunction with the 9th ACM Conference on Bioinformatics, Computational Biology, and Health Informatics (ACM BCB) (http://acm-bcb.org/2018/) on August 30th, 2018. This supplement collects the system descriptions of top-performing solutions of the tasks.

## Task 1: family history extraction

As a risk factor of many diseases, family history information (FHI) captures shared genetic variations among family members [2]. Information such as age, gender, and degree of relatives are also considered when taking into the account of risk assignment of a large number of common diseases. The fact that many care process models use FHI highlights the importance of FHI in the decision-making process of diagnosis and treatment. However, extracting accurate and complete FHI from clinical texts remains challenging as a clinical NLP problem due to the lack of standardized evaluation mechanisms and publicly available language resources.

To curate a corpus that can be made publicly available without losing semantic power for potential information extraction systems, we first collected the clinical narrative from family history sections of clinical notes at Mayo Clinic Rochester, the content of which are highly relevant to FHI. A team of annotators annotated the original corpus with clinical observations, family member mentions and protected health information. Afterwards, the protected health information is replaced with synthetic yet meaningful strings, and the clinical observations, family member mentions are shuffled among the corpus to further protect patient privacy.

Leveraging the synthetic corpus with FHI, we organized this shared task to encourage the community to propose and develop family history extraction (FHE) systems [3]. The task composes two subtasks. The Subtask 1 focuses on identifying family member entities and clinical observations (diseases), and the Subtask 2 expects the association the living status, side of the family and clinical observations to family members to be extracted. The Subtask 2 is an end-to-end task which is based on the result of the Subtask 1. A total of 5 teams submitted overall 14 submissions for the official evaluation, and the descriptions of 2 teams are included in this supplement.

The solution proposed by Dai focused on the extraction step and formulates it as a sequence labeling task. A neural sequence labeling model along with different tag schemes to distinguish family members and FHI-related observations was developed. Corresponding to different tag schemes, the identified entities were aggregated and processed by different algorithms to determine the required properties. The effectiveness of encoding required properties in the tag schemes was evaluated by the task corpus. The developed neural network-based models performed significantly better than the conditional random fields models.

Shi et al. explored two joint learned models for the two subtasks. For the entity extraction subtask, the Bidirectional Long Short Term Memory (Bi-LSTM) and Conditional Random Field (CRF) models are used to recognize FHI related entities using word embeddings and part-of-speech (POS) embedding as inputs. For the relation extraction subtask, they trained a Bi-LSTM to classify the relations. The two models are joint trained towards a customized loss function to combine the loss from the two subtasks. On top of the results from machine learning models, they used heuristic rules and post-processing to handle entity properties such as side of family and living status.

## Task 2: clinical semantic textual similarity

The frequent use of copy-and-paste, templates, and smart phrases have resulted in redundant texts in clinical notes, which may reduce the EHR data quality and add cognitive burden of tracking complex records in clinical practice. Therefore, there is a growing need for tools that can aggregate data from diverse sources and minimize data redundancy, and organize and present the EHR data in a user-friendly way to reduce physicians’ cognitive burden. One technique for automatically reducing redundancy in free text EHRs is to compute semantic similarity between clinical text snippets and remove highly similar snippets. Semantic textual similarity (STS) is a common task in the general English domain to assess the degree to which the underlying semantics of two segments of text are equivalent to each other. The assessment is usually performed using ordinal scaled output ranging from complete semantic equivalence to complete semantic dissimilarity. The STS task has been held annually since 2012 to encourage and support research in this area. However, these series of STS tasks used texts in the general English domain and no STS shared task focuses on the text data in the clinical domain. To motivate the biomedical informatics and NLP communities to study STS in the clinical domain, we initiated the ClinicalSTS task to provide a venue for evaluation of the state-of-the-art algorithms and models.

ClinicalSTS provides paired clinical text snippets for each participant. The corpus, named MedSTS, consists of deidentified clinical sentences from narrative clinical notes [4]. The participating systems were asked to return a numerical score indicating the degree of semantic similarity between the pair of two sentences. Performance is measured by the Pearson correlation coefficient between the predicted similarity scores and human judgments. The scores fall on an ordinal scale, ranging from 0 to 5 where 0 means that the two clinical text snippets are completely dissimilar (i.e., no overlap in their meanings) and 5 means that the two snippets have complete semantic equivalence.

Xiong et al. proposed a novel framework based on a gated network to fuse distributed representation and one-hot representation of sentence pairs. Some current state-of-the-art distributed representation models, including Convolutional Neural Network (CNN), Bi-LSTM and Bidirectional Encoder Representations from Transformers (BERT), were used in their system. Compared with the systems only using distributed representation or one-hot representation, their proposed method achieved higher performance. Among all distributed representations, BERT performed best. Further analysis indicates that the distributed representation and one-hot representation are complementary to each other and can be fused by gated network.

Chen et al. demonstrated both their participating systems and improvements after the challenge. They applied sentence embeddings pre-trained on PubMed abstracts and MIMIC-III clinical notes and updated the Random Forest and the Encoder Network. During the challenge task, no end-to-end deep learning models had better performance than machine learning models that take manually-crafted features. In contrast, with the sentence embeddings pre-trained on biomedical corpora, the Encoder Network now achieves higher performance than the original best model. The ensembled model taking the improved versions of the Random Forest and Encoder Network as inputs further improves the performance. Deep learning models with sentence embeddings pre-trained on biomedical corpora achieve the highest performance on the test set. Error analytics indicates that end-to-end deep learning models and traditional machine learning models with manually-crafted features can complement each other, which suggests that a combination of these models can better find similar sentences in practice.

## References

[1] Blumenthal D (2011). Implementation of the Federal Health Information Technology Initiative. N Engl J Med.

[2] McCarthy JJ, Mendelsohn BA (2016). Family history. In: precision medicine: a guide to genomics in clinical practice.

[3] Liu S, Rastegar-Mojarad M, Wang Y (2018). Overview of the BioCreative/OHNLP 2018 family history extraction task. BioCreative/OHNLP 2018 Workshop Proceedings.

[4] Wang Y, Afzal N, Fu S, et al. MedSTS: a resource for clinical semantic textual similarity. Lang Resour Eval. 2018. 10.1007/s10579-018-9431-1.

